# Significant Clinical Manifestations in Ballantyne Syndrome, after a Case Report and Literature Review: Recognizing Preeclampsia as a Differential Diagnosis

**DOI:** 10.1155/2019/2013506

**Published:** 2019-03-05

**Authors:** Silvia F. Navarro-Perez, Karen Corona-Fernandez, José L. Rodriguez-Chavez, A. Bañuelos-Franco, María G. Zavala-Cerna

**Affiliations:** ^1^Unidad de Investigación en Ginecología y Alto Riesgo Obstétrico, División de Ginecología y Obstetricia del Hospital General de Occidente, Av. Zoquipan #1050, Col. Seattle, 45170, Mexico; ^2^Immunology Research Laboratory, International Program of Medicine, Universidad Autonoma de Guadalajara, 44100, Mexico; ^3^Unidad de Investigación en Ginecología y Alto Riesgo Obstétrico, División de Ginecología y Obstetricia del Hospital General de Occidente, Mexico

## Abstract

Ballantyne syndrome (BS) also called mirror syndrome is defined by the presence of a clinical triad that includes fetal hydrops and placental and maternal edema. Here we present a clinical case of a 34-year-old woman with a 29 weeks' pregnancy, who developed BS and fetal loss probably due to failure in prompt recognition of a rapidly growing sacrococcygeal teratoma (SCT). Due to similarities in clinical presentation with preeclampsia and the importance in early identification of the source for BS, we underwent a literature review in order to identify significant signs and symptoms, as well as sonographic changes, in order to help clinicians to make this prompt recognition, identification of the cause, and early management of BS, which will have an important impact in maternal and fetal survival.

## 1. Introduction

 Ballantyne syndrome (BS) also called mirror syndrome or triple edema is defined by the presence of a clinical triad that includes fetal hydrops and placental and maternal edema [1]. The term mirror as the name implies is due to an edema that is mirrored in both, the fetus and the mother [2]. John William Ballantyne in 1892 was the first to acknowledge the syndrome, originally thought to be the consequence of rhesus isoimmunization; however currently more than 80 different causes have been described and not all of them have an immunological origin [3], with only a few reported cases [4]. The syndrome appears in one out of 3,000 pregnancies; however the incidence can be underestimated, due to similarities in clinical presentation with preeclampsia (PE) [1, 3], which include maternal edema (80-100%), hypertension (60%), proteinuria (20-56%), anemia (46%), rapid weight gain, progressive dyspnea, and albuminuria [5]. Unlike preeclampsia in BS patients are hemodiluted according to blood indices with a typical low hematocrit [6]. In addition to similarities in their clinical presentation, preeclampsia and BS can coexist and compromise to a greater extent pregnancy resolution, as well as the maternal and fetal prognosis. In preeclampsia the recognition of abnormal placentation with defective spiral artery remodeling, favoring progressive placental ischemic reperfusion and excessive oxidative stress has been clearly established [7]. These changes result in a pathological imbalance among angiogenic (VEFG) and anti-angiogenic circulating factors (sFIt, activin) [8]. On the contrary, the etiology and pathogenic mechanisms in BS are still partially described and remain unclear, probably due to a limited number of papers [9]. Some factors that have been previously associated with the development of BS include diverse conditions, such as nonstructural causes: rhesus isoimmunization, fetal supraventricular tachycardia, vertical cytomegalovirus, parvovirus B19 and Coxsackie virus infection, and laser photocoagulation for twin-to-twin transfusion syndrome, as well as structural alterations: aneurysm of Galen's vein, sacrococcygeal teratoma (SCT), or placental chorioangioma [10, 11]. 

It is extremely important for clinicians to identify similarities and differences between BS and preeclampsia, since diagnosis and management of BS might be possible when the source is readily identified [3]. Here, we present a case report of BS probably secondary to SCT that was not promptly identified. Additionally, we underwent a literature review to identify clinical similarities and differences between preeclampsia and BS, in order to provide hallmarks for prompt recognition and treatment of the base pathology that is responsible for BS, which will decrease maternal and newborn fatalities.

## 2. Case Report

**Figure 1:**
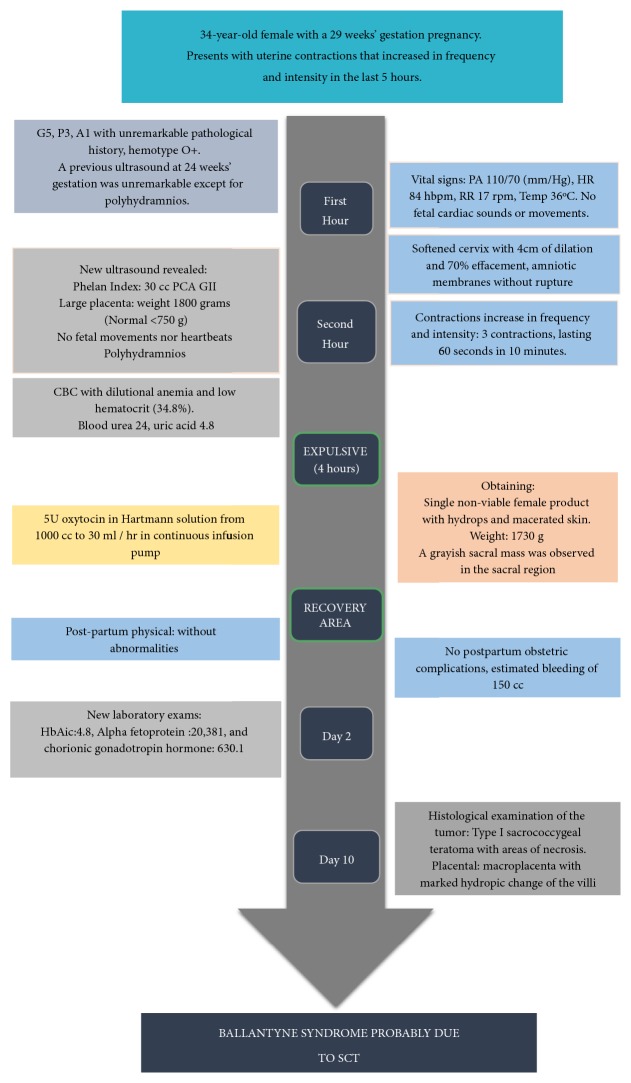
Timeline of patient's clinical evolution with diagnostic tests and treatments. SCT: sacrococcygeal teratoma.

**Figure 2:**
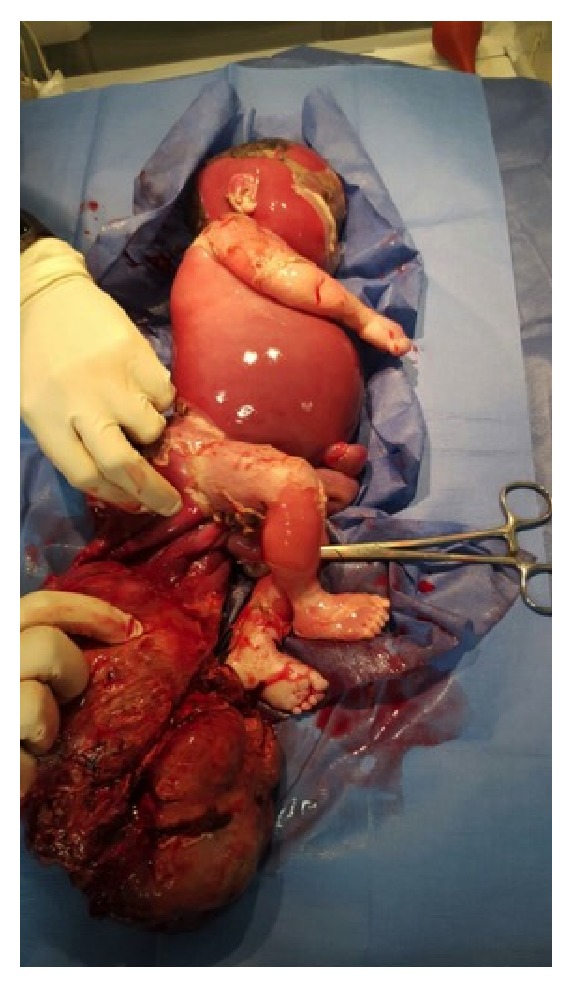
Single female sex without vitality spontaneously delivered, with hydrops and macerated skin, weight: 1730 g, with a grayish and hemorrhagic sacral mass, later described as a Type I sacrococcygeal teratoma (SCT).

A 34-year-old woman gravida 5, para 3, abortion 1 presented to the ER of our hospital, at 29 weeks' gestation, due to uterine contractions that increased in frequency and intensity in the last 5 hours, with no other symptomatologies added (Figure 1). Her past medical history was unremarkable with O + hemotype; she had an abortion due to an anembryonic pregnancy that required curettage, which was performed without complications. The patient had 3 healthy previous pregnancies which resulted in 3 healthy living children. Currently in her fifth pregnancy, she denies pregnancy care; only one obstetric ultrasound performed at 24 weeks' gestation in another clinic reported the following: harmonic fetal growth and no fetal malformations; however polyhydramnios was present. During her observation in the ER, a new ultrasound examination was ordered, which revealed an apparently large placenta with approximate weight of 1,800 gr, suggestive of placental edema; the fetus appeared with polyhydramnios, and no heartbeats nor fetal movements were registered; she was then referred to the high risk obstetric department, where she was found to have normal vital signs, mild edema of the ankles without fovea, and a gravid uterus occupied by a single longitudinal cephalic fetus with lateralized back to the left; fetal heart rate was not detected with doptone; she had regular uterine dynamics palpable at a rate of 3 contractions lasting 60 seconds each, within a time frame of 10 minutes; at vaginal examination the cervix was softened with 4 cm of dilation and 70% thinned, intact amniotic membranes, without bleeding or leucorrhoea. Laboratory tests reported the following: hemoglobin 11.5 g/dl, hematocrit 34.8%, and no other abnormal results including normal renal and hepatic function. Due to increased frequency of contractions, she was immediately sent to the expulsive room, where a single female sex without vitality was spontaneously delivered, with data of hydrops and macerated skin, weight: 1,730 gr, with a grayish and hemorrhagic sacral mass (Figure 2). After resection of the tumor and pathologic examination the following report was made: An 820 gr Type I sacrococcygeal teratoma (SCT) with mature and immature elements as well as blood sequestration areas, partially encapsulated with extensive areas of coagulation and necrosis. Placenta was grossly edematous and weighed 1,280 gr with a marked hydropic change of the villi, with an area of intervillous fibrinoid infarct was noticed. Amnion and chorion had no alterations. The umbilical cord had three blood vessels with marked edema of Wharton's jelly. The patient received counseling and was discharged 3 days after delivery without edema or any other physical alteration.

## 3. Discussion

In the present case, the diagnosis of BS was established after the findings of fetal hydrops, placental edema, and mild maternal edema. Presumably in this case, BS developed secondary to SCT. The SCT was not evident in the first ultrasound at 24 weeks' gestation, neither were fetal hydrops; however polyhydramnios was present at that time. SCT is a rare congenital disease of the newborn that happens in 1 out of 20,000-40,000 pregnancies, predominantly in female newborns with a ratio of 3:1 [12]. The perinatal mortality of SCT has been estimated from 40 to 50% [13]. Death occurs in fetuses with fast growing, solid, and highly vascularized teratomas which can originate high-output cardiac failure, which is a consequence of the tumor acting as a large arteriovenous malformation that steals vascular supply from the fetus [14]. Goto et al. published the report of two cases of SCT in which only one was associated with BS and a fetal outcome for the fetus [15].

The etiopathogenic mechanisms of BS remain partially understood; it has been suggested that the placenta is the source of the problem, since resolution of the pregnancy with the removal of the placenta resolves the mirroring edema [16]. Some previous reports have stated that compensatory mechanisms originate as a consequence of placental ischemia and are responsible for an increased placental blood flow with a rise in placental renin up to 10-fold, which will aid in increased maternal aldosterone a fluid retention [17]. Furthermore, some molecules have been identified in association with endothelial dysfunction in BS, such as Activin A and follistatin; other molecules are involved in inflammation and increased vascular permeability including ICAM-1 and vWF; the potent vasoconstrictor ET-1 [10]; and the anti-angiogenic factor sVGEFR-1 [18]. Nevertheless, it would be of interest to determine the abnormalities in the fetus that will later give rise to these alterations in the placenta that finally manifest in BS, which is a life threatening condition for the mother and the fetus [19].

In the fetus, the development of hydrops secondary to SCT can be explained by a high output failure caused by either anemia due to tumor hemorrhage and/or an arteriovenous shunt in a low resistance, rapidly growing tumor [20, 21]. Cardiac failure can give rise to an alteration in the regulation of the fluid dynamics between vascular and interstitial spaces, with an imbalance of interstitial fluid production and lymphatic return, which will eventually lead to fetal hydrops [19]. The fetus is particularly susceptible to interstitial fluid accumulation because of its greater capillary permeability compliant interstitial compartments and vulnerability to venous pressure on lymphatic return. Compensatory mechanism to maintain homeostasis during hypoxia results in increased venous pressure and interstitial fluid accumulation, developing the characteristic hydropic changes [22]. In our case the histologic examination of the tumor demonstrated signs of bleeding; nevertheless the presence of an arteriovenous shunt was not examined. The presence of fetal hydrops in our case was evident at delivery, established by accumulation of fluid in the skin and abdomen (Figure 2), in accordance with fetal hydrops definition [23].

Furthermore, rapid growth of SCT (>150 cm^3^ per week) and hypervascularity are associated with increased risk in perinatal mortality [12]. A previous study stated that SCT bigger than 5 cm can be involved in the formation of high volume arteriovenous shunts that can lead to cardiac failure, placentomegaly, hydrops, and preeclampsia or BS [14]. This seems to be in accordance with our case; although the growth of the tumor was not registered by week, it was evident that most of the growth happened after 24 weeks' gestation and before 29 weeks' gestation.

*Differential Diagnosis. *When establishing a differential diagnosis, it is important to acknowledge that the most common complications in pregnancy are hypertensive disorders such as preeclampsia with an estimated prevalence of 10-12% worldwide; nevertheless it may coexist with other pathologies including BS. It is yet to be determined whether the treatment of the underlying cause of BS could improve preeclampsia as well.

In order to distinguish BS from preeclampsia, one important variable is time for presentation since BS can present earlier, with the first becoming evident as soon as 16 weeks' gestation and the latter presenting after 20 weeks' gestation [1]. Please refer to Table 1 in order to compare this and other considerations for each pathology.

Maternal rapid weight gain, marked skin edema, and uterine distention can also be distinguishable features in BS, whereas elevated blood pressure and proteinuria can be present in both [11]. Although maternal edema is an important clinical consideration, our case presented with only mild maternal edema; we found only few cases where maternal edema could be absent or mild in BS secondary to SCT [11].

Renal failure can appear in both; however, it has been described in a higher frequency for BS; postpartum hemorrhage and pulmonary edema have also been described in BS [24]. Other unspecific findings common to both include headache, nausea, vomiting, dyspnea, oliguria, pulmonary edema, anemia, elevated uric acid/creatinine, and elevated liver enzymes [25]. Laboratory findings of special interest for differential diagnosis are a dilutional anemia with low hematocrit, thrombocytopenia, and low serum albumin which are characteristic of BS, whereas in preeclampsia patients typically present with hemoconcentration [1, 26]. Dilutional anemia and thrombocytopenia in BS might be explained through the finding of high maternal serum vasopressin and atrial natriuretic factor [11].

First-trimester uterine artery Doppler ultrasound examination, placental growth factor, and pregnancy-associated plasma protein-A help to predict pregnancy complications associated with uteroplacental insufficiency such as preeclampsia before the onset of clinical features [27]. Also, during ultrasound examination in BS the most common fetal findings are hydrops fetalis (involving peritoneal compartment, cutaneous compartment, pleural space, pericardial space, and fetal scalp), polyhydramnios, placental edema, fetal anomalies, organomegaly, or a tumor, alone or simultaneously [1].

Development of BS can increase the risk of preeclampsia and vice versa; we believe that there is a common ground for the development of both pathologies, a ground that favors an angiogenic-antiangiogenic imbalance that might contribute to an even worse prognosis when both entities coexist; therefore it is mandatory to alert physicians about this possibility in order to remain vigilant and prescribe early treatment when indicated.

*Treatment. *BS syndrome should be treated accordingly to the source or the reason for the endothelial dysfunction. Placental or fetal malignancies are rare complications during pregnancy, but when they occur, they may present significant challenges. In our case SCT was identified as the possible source for BS. The importance in this association is that SCT diagnosed prenatally can be managed to avoid fetal fatalities [14]. Fetal surgery of SCT is a viable option for treatment in selected patients that demonstrate signs of cardiovascular decline during gestation, or when the mother is at risk [28]. Beyond 27 weeks' gestation, however, the risk for the development of complications related to the surgery increases significantly; therefore early delivery (27 to 32 weeks' gestation) with immediate SCT resection has been shown to be a better option than “watchful waiting” documented by Baumgartner et al. with a survival rate of 81.8% [13]. Important considerations for the success of earlier delivery include a low compromised hydropic fetus, absence of high-output cardiovascular failure, absence of tumor bleeding or fetal anemia, and having appropriate facilities for neonatal intervention and immediate surfactant administration [13].

Maternal complications of BS, such as hypertension and pulmonary edema, should be immediately treated if there is a decision to wait with continuous observation and the administration of diuretics, calcium channel blockers, and beta-blockers [29].

## 4. Conclusions

We presented a case report with BS probably following to the development of SCT after 24 weeks' gestation, with polyhydramnios, hydrops fetalis, placental edema, and mild maternal edema. Since the most common cause for the development of hydrops fetalis is preeclampsia, we thought it could be important to identify clinical differences that could help in a prompt diagnosis since management of both entities is different, especially in BS where the source for endothelial dysfunction can be recognized prenatally. Preeclampsia and BS share some similarities in clinical presentation; even more, they can occur simultaneously, increasing the risk of maternal and fetal death. Some clinical considerations that point towards BS in the mother are time of onset (<20 weeks), edema, renal affection, and hemodilution; in the fetus ultrasound examination: polyhydramnios, cardiac affection, and fetal anomalies, including tumors. Finally, we would like to emphasize that in our case the initial manifestation was polyhydramnios; therefore, it is mandatory that patients with this abnormality are adequately observed during the following weeks' gestation with a Doppler ultrasound or fetal MRI for the evaluation of hydrops fetalis, and, in the case of a tumor, growth rate and vascularity. Then, each case should be evaluated for either early termination of pregnancy or fetal surgical intervention, in order to decrease the risk of fetal mortality, as well as maternal morbidity and mortality associated to BS.

## Figures and Tables

**Table 1:** Clinical considerations in establishing a differential diagnosis for BS.

Variable	PE	BS	Case Report
Maternal			

Gestational age	< 20	16-34	24-29

Edema	++	++	+

Hypertension (> 130/80)	+++	+	-

Proteinuria (> 300mg/day)	+++	+	+

Hyperreflexia (OTR)	+	-	-

Liver function test abnormalities	+++	+	+

Creatinine	++	+	+

Urea	++	+	+

Creatinine:urea ratio	++	+	+

Thrombocytopenia	+++	+	-

Uterine bleeding	++	+	-

Renal failure	++	+++	-

Pulmonary edema	+	+++	-

Anemia	Hemoconcentration	Hemodilution	Hemodilution

Placental edema	-	+++	+++

Fetal			

Hydrops	+	+++	+++

Polyhydramnios	+	+++	+++

Cardiac affection	+	++	Unknown

Intrauterine growth restriction	+	-	-

Associated tumor	-	+	+

Fetal death	+	++	++

## Additional Points

*Highlights*. (i) Ballantyne syndrome and preeclampsia have different etiologies but similar clinical presentations; (ii) identification of significant clinical differences between both will contribute to accurate prompt diagnosis; (iii) prompt treatment of BS will have a beneficial impact in maternal and fetal survival.
